# Dissection-independent production of *Plasmodium* sporozoites from whole mosquitoes

**DOI:** 10.26508/lsa.202101094

**Published:** 2021-06-16

**Authors:** Joshua Blight, Katarzyna A Sala, Erwan Atcheson, Holger Kramer, Aadil El-Turabi, Eliana Real, Farah A Dahalan, Paulo Bettencourt, Emma Dickinson-Craig, Eduardo Alves, Ahmed M Salman, Chris J Janse, Frances M Ashcroft, Adrian VS Hill, Arturo Reyes-Sandoval, Andrew M Blagborough, Jake Baum

**Affiliations:** 1 Department of Life Sciences, Imperial College London, Sir Alexander Fleming Building, London, UK; 2 The Jenner Institute, University of Oxford, Old Road Campus Research Building, Oxford, UK; 3 Department of Physiology, Anatomy and Genetics, Henry Wellcome Building for Gene Function, University of Oxford, Oxford, UK; 4 Medical Research Council London Institute of Medical Sciences, Imperial College London, Hammersmith Hospital, London, UK; 5 Department of Parasitology, Leiden Malaria Research Group, Center of Infectious Diseases, Leiden University Medical Center, (LUMC, L4-Q), Leiden, The Netherlands; 6 Instituto Politécnico Nacional, Mexico City, Mexico; 7 Department of Pathology, University of Cambridge, Cambridge, UK

## Abstract

Progress towards a protective vaccine against malaria remains slow. To date, only limited protection has been routinely achieved following immunisation with either whole-parasite (sporozoite) or subunit-based vaccines. One major roadblock to vaccine progress, and to pre-erythrocytic parasite biology in general, is the continued reliance on manual salivary gland dissection for sporozoite isolation from infected mosquitoes. Here, we report development of a multi-step method, based on batch processing of homogenised whole mosquitoes, slurry, and density-gradient filtration, which combined with free-flow electrophoresis rapidly produces a pure, infective sporozoite inoculum. Human-infective *Plasmodium falciparum* and rodent-infective *Plasmodium berghei* sporozoites produced in this way are two- to threefold more infective than salivary gland dissection sporozoites in in vitro hepatocyte infection assays. In an in vivo rodent malaria model, the same *P. berghei* sporozoites confer sterile protection from mosquito-bite challenge when immunisation is delivered intravenously or 60–70% protection when delivered intramuscularly. By improving purity, infectivity, and immunogenicity, this method represents a key advancement in capacity to produce research-grade sporozoites, which should impact delivery of a whole-parasite based malaria vaccine at scale in the future.

## Abstract (precis)

Overcoming limitations of traditional dissection-dependent methods for malarial sporozoite isolation from mosquitoes, a new dissection-free method is presented that yields pure, infective sporozoites.

## Introduction

A vaccine against malaria is still urgently required to address the nearly half a million deaths caused by the disease each year (1). Developmental malaria vaccines currently focus on two distinct strategies, either recombinant production and immunisation of dominant surface antigens from the *Plasmodium* parasite, or labour-intensive production, purification, and immunisation with live parasites that are attenuated or delivered under drug coverage (2). The most advanced of these vaccines have focussed on preventing the pre-erythrocytic stages of parasite infection, targeting the infectious sporozoite form between its injection into the skin by the feeding mosquito and its first destination within the human liver (2). Pre-erythrocytic subunit vaccines to date have largely been based on the dominant sporozoite surface antigen, circumsporozoite protein (CSP) (3). CSP has been the subject of intensive investigation for more than 40 yr. Its most recent formulation within the RTS,S vaccine confers moderate protection following challenge in phase 3 trials, although long-term efficacy remains uncertain (4). Sporozoite-based vaccination by controlled mosquito-bite with drug coverage on the other hand, has consistently been shown to confer sterile (complete) protection (5). Mosquito-based delivery for large-scale vaccination is, however, impractical. The best alternative to this is live-attenuated sporozoite vaccination delivered directly (6, 7, 8, 9, 10). By producing parasites that arrest prematurely in the liver (11, 12), both humoral and cellular immunity can develop, offering long-term protection (8, 13). Indeed, the most advanced of these, called PfSPZ (*P. falciparum* sporozoite) (14), can confer sterile protection under controlled clinical conditions. A limitation to its utility remains the requirement for i.v. delivery and use of substantial sporozoite numbers, as high as 270,000 sporozoites per immunisation, and the requirement for multiple rounds of immunisation (typically one prime and three boosts) (2, 8, 13). PfSPZ has shown moderate efficacy (up to 52% at 24 wk) against naturally transmitted malaria (15), although vaccine efficacy long-term is still pending. Thus, while showing great promise PfSPZ, such as RTS,S, still falls short of the preferred efficacy aspired to for future malaria vaccines (16).

Live-attenuated whole sporozoite vaccination approaches rely on generating large amounts of pure and aseptic parasites for clinical grade vaccine manufacture (13). This is a considerable bottleneck to vaccine design, testing, and implementation in terms of scale, time, and cost. At present, sporozoites can only be isolated from infected mosquitoes via salivary gland dissection (SGD). As well as the obvious challenges this presents to vaccine development, difficulties with sporozoite isolation have also held back general understanding of sporozoite biology. While substantial advances have been made in development of experimental in vitro hepatocyte models of infection (17, 18), these are much less accessible and less reproducible when compared to routine culture and study of blood-stage parasites. SGD requires in vivo parasite development in the mosquito followed by manual dissection of the salivary glands 15–21 d post infected blood feed. Originally described in 1964 (19) with only minor variations since (17, 20, 21, 22), the dissection method involves mosquito decapitation, gland removal and homogenisation to release sporozoites. Dissection in this way is time-consuming, taking a skilled technician an hour or so to dissect 100 or more glands to a reasonable standard. With total extraction time being a critical factor for subsequent sporozoite viability (23), there is a relatively low upper limit for attaining live, infectious sporozoites. Furthermore, SGD sporozoites retain a considerable amount of mosquito-originating debris (24). Some of this debris, for example, salivary gland–associated proteins, have been shown to inhibit sporozoite motility, which is critical for liver cell infection (25). Other mosquito-derived contaminating proteins have been shown to modulate immune response in vivo (26), potentially affecting vaccinations. Likewise, the time taken, and contamination carried over, places limits on the infectivity and development of *Plasmodium* sporozoites in vitro (20, 24, 27). Rates of cell infection with in vitro hepatocyte (primary or hepatoma) cultures using SGD are typically <1% using the rodent malaria model *Plasmodium berghei* (28, 29, 30, 31) and <2% for human *Plasmodium falciparum* sporozoites (18, 20, 22, 24, 32, 33). These limitations have been a major impediment to in vitro studies and for screens that rely on high rates of infection.

Several previous attempts have sought to improve throughput and purity of whole sporozoite preparation. Methods aimed at bypassing SGD have included centrifugation through glass wool (34) and compression between glass plates (35). These alternative methods have not substantially improved parasite purity, even when combined with density gradients (33, 36, 37, 38, 39, 40). While the addition of gradients increase sporozoite yield, the final output is still contaminated with mosquito debris (25, 41). Other methods trialled for sporozoite isolation have included ion exchange chromatography (41, 42), and later free-flow electrophoresis (FFE) (43). FFE is a liquid form of electrophoresis commonly used to separate organelles under native conditions based on net surface charge (44). The poor yields or complexity of these two methods has limited interest in their scaled usage. This is despite significant, although unexplored, recent developments in FFE technology in particular (http://www.ffeservice.com). To date, the only scaled means for manufacture of a clinical grade vaccine has therefore relied on enlisting multiple skilled manual dissectors combined with rearing of parasites within aseptic mosquitoes. Automated aids for dissection have been described, although these still require some manual mosquito manipulation (45 *Preprint*).

Obtaining malaria sporozoites therefore remains a major challenge for improving understanding of basic parasite transmission biology, and a significant hurdle for scalable and reproducible production of whole sporozoites for direct vaccination. In response to this challenge, here we describe a multi-step method that successfully purifies both rodent *P. berghei* and human *P. falciparum* sporozoites from whole mosquitoes in a batch process, based on an optimized combination of homogenisation, size exclusion, density and charge. This stepwise approach facilitates processing of hundreds of mosquitoes per hour, rapidly harvesting pure sporozoites, and can be adapted to produce effectively contaminant-free, vaccine-grade sporozoites by a single trained technician. The sporozoites isolated by this process show excellent infectivity both in vitro and in vivo and offer sterile protection in a rodent model when given as a live-attenuated vaccine or 60–70% protection when administered via intra-muscular delivery. Being dissection-independent, this process can facilitate the rapid and scalable manufacture of *Plasmodium* sporozoites to advance pre-erythrocytic *Plasmodium* research and as an enabling technology that can be harnessed for delivery of future whole-parasite based malaria vaccines.

## Results

### Rapid, dissection-independent, isolation of sporozoites from whole mosquitoes

**Figure 1.**
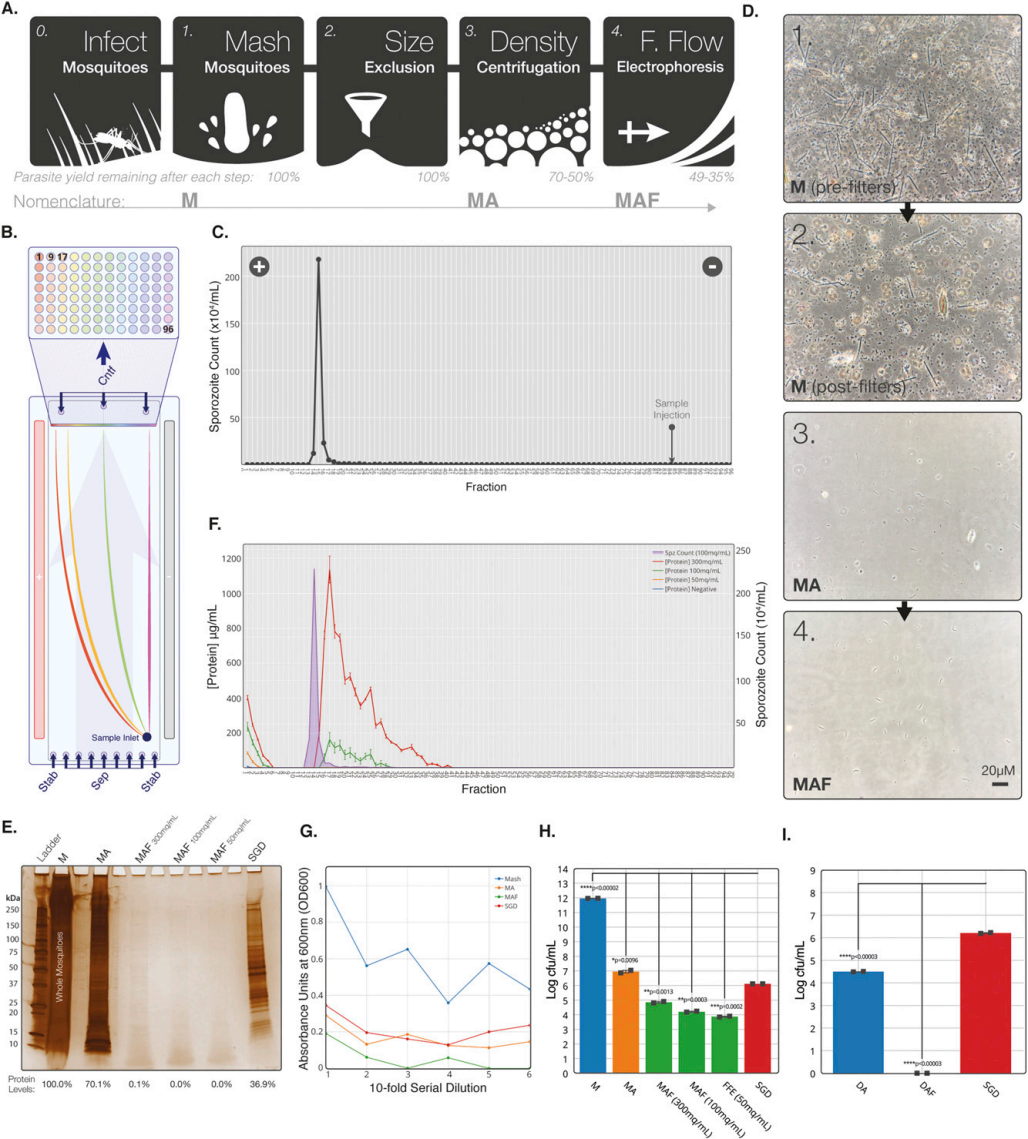
Development of a stepwise process for purification of sporozoites from whole mosquitoes. **(A)** Schematic of key steps in the sporozoite purification process. **(B)** Schematic representation of sample separation by continuous zone electrophoresis mode. An electrophoretic buffer is run through a chamber 0.5 mm thick with a voltage applied across the flow. Sample added to the start of the chamber is carried vertically up the length of the chamber (pale blue arrow) as a voltage is applied across, separating across the horizontal length of the chamber. The outflow from the chamber is separated into 96 outlets along the horizontal length of the chamber, which drop into a 96 well plate. **(C)** Manual sporozoite count by haemocytometer of free-flow electrophoresis (FFE) fractions from a representative MAF sporozoite separation. Point of sample injection indicated by arrow and direction of current indicated by positive and negative symbols. **(D)** Bright-field images of each stage of purification from whole mosquito homogenate. All stages diluted to 7 × 10^5^ sporozoites/ml. **(E)** Silver stain of reducing SDS–PAGE gel with uninfected mosquitoes (four MEQs) from each step of purification. Uninfected MAF lanes are from the same fraction as the sporozoite peak fraction identified by running infected mosquitoes at the same time. **(F)** Protein concentration in each fraction after loading uninfected mosquito MA onto the FFE machine at three doses of mosquitoes (MAF). Sporozoite distribution (purple) from infected mosquitos loaded at 100 mq/ml is marked to allow comparison of purification. **(G)** End point 16-h serial dilution for each step of MAF purification. Absorbance of samples in TBS was measured at 600 nm (OD600) 16 h post-inoculation ay 37°C. All growth conducted at 37°C, 17*g*, using mosquitoes blood-fed on uninfected mice 21 d before MAF extraction. **(H)** Bacterial growth (samples normalised to MEQ of 200 mq/ml) at different stages from uninfected whole mosquito (M) origin purification. Samples were loaded onto the FFE machine at three different originating mosquito doses. **(I)** Bacterial growth (samples normalised to MEQ of 200 mq/ml) at different stages from infected SGD-origin purification. Experiments show the mean of two technical replicates and error bars represent SEM. All treatments compared with dissected by unpaired two-tailed *t* test using Bonferroni correction (H: *P < 0.01, **P < 0.002, ***P < 0.0002, ****P < 0.00002; I: *P < 0.017, **P < 0.003, ***P < 0.0003, ****P < 0.00003).

The challenges of obtaining sporozoites for malaria research by SGD are a major impediment to improving understanding of the liver stages and development of effective whole-parasite vaccination (2, 24, 30, 31, 32, 46, 47, 48, 49). Sporozoite isolation by SGD is a low-throughput and labour-intensive procedure which produces sporozoites of mixed purity, often contaminated with mosquito-associated material. To address this need, we have developed a stepwise method for the purification of sporozoites from whole mosquitoes without requiring manual dissection (Fig 1A).

**Figure S1.**
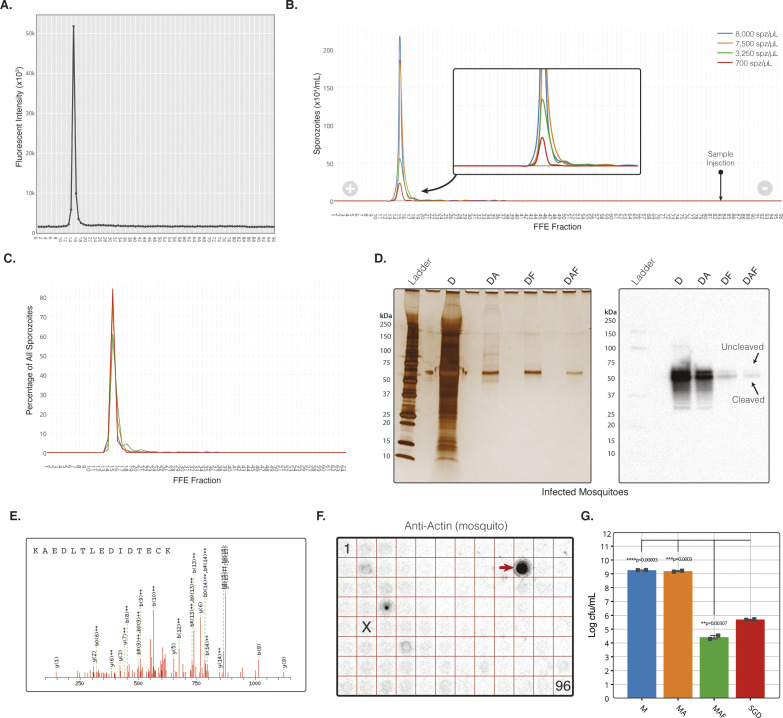
The MAF Purification platform additional data. **(A)** Fluorescent plate read at 610 emission of free-flow electrophoresis (FFE) fractions from a representative MAF sporozoite separation. **(B)** Parasite distribution into FFE fractions when loaded at four different sporozoite doses (spz/ml). Quantification by haemocytometer count. **(C)** Sporozoite distribution based on total percent of sporozoites per fraction for each sporozoite dose. **(D)** Left; silver stain of infected mosquitoes from each step of purification when using dissected salivary glands for homogenisation instead of total mosquitoes. Right; Western blot against *Plasmodium berghei* CSP from the same samples as the silver stain (left). MAF samples in (D) where injected into the FFE machine at 100 mq/ml. All silver stains from reducing SDS–PAGE’s with samples normalised by MEQ with four MEQs loaded onto each lane. After MAF purification, the ratio of uncleaved:cleaved was 1:7.8, compared with 1:1.8 for salivary gland dissection sporozoites (assessed by densitometry). Cleaved and uncleaved bands indicated by arrows, with expected sizes of 45 and 55 kD, respectively. **(E)** Liquid chromatography tandem mass spectrometry (LC-MS/MS) analysis of each stage of purification. LC-MS/MS raw data searched against the Uniprot-Swissprot database using the MASCOT search algorithm identified *P. berghei* CSP protein exclusively with three peptides in the fraction purified by MAF only. Identification of tryptic peptide 297–312 from *P. berghei* CSP protein (Swissprot ID: P06915) with MASCOT score of 37 by mass spectrometry analysis. All samples normalised to 200 mq/ml. **(F)** Dotblot of all 96 FFE fractions in a plate layout against mosquito actin protein (A2066; Sigma-Aldrich). Actin positive fraction indicated by arrow. Peak fraction containing most sporozoites is indicated by “X,” Fraction 1 and 96 indicated. **(G)** Bacterial growth at different stages from infected whole mosquito (M) origin purification.

Our method consists of three discrete steps, with capacity to process up to 1,000 mosquitoes at a time (over a 2-h window) by a single individual. Whole mosquitoes were homogenised to release sporozoites and filtered sequentially through 100–10-μm filters. The filtered mosquito homogenate/Mash (M) was then pre-purified by density centrifugation using Accudenz (MA), as previously described (24), to remove larger mosquito-associated debris from sporozoites. The sporozoite layer was subsequently purified by FFE, based on total net charge (MAF) (Fig 1B and Table S1) using a continuous zone electrophoresis (cZE) mode (see the Materials and Methods section). Output consisted of 96 fractions with a peak sporozoite fraction, as represented by purification of rodent malaria *P. berghei* mCherry sporozoites assessed by light microscopy (Fig 1C) or fluorescent plate reader for mCherry fluorescence (Fig S1A).

Table S1 Abbreviations.

*P. berghei* sporozoites produced according to this multi-step method demonstrated reproducible separation, independent of initial sporozoite quantity. Most sporozoites separated into a single fraction, with a characteristic tail in the distribution that lengthened as sporozoite dose load increased (Fig S1B and C). This peak fraction was used for all subsequent experiments. A single run resulted in a final 51–65% loss in yields compared with the starting material; however, the FFE step was responsible for only a 30% loss of sporozoites from the preceding step in the protocol. The Accudenz step was responsible for up to a 50% loss in yield from its preceding step. Of note, Accudenz on its own is known to be associated with a loss of sporozoite yield of up to 50%. Using this method, ∼500–1,000 mosquitoes could be processed by one individual in 2 h. This same skilled user could process a few hundred mosquitoes in a similar timeframe using traditional SGD (range of 100–200 by our experienced dissectors). This represents a 5–10-fold increase in throughput comparing MAF to SGD (incorporating Accudenz purification) with the potential for running parallel units to scale production accordingly, although without requiring additional staffing.

Given the established potential of whole sporozoites as an effective vaccine (9), we next sought to establish the purity of MAF sporozoites compared with SGD sporozoites. Initial assessment of bright-field images showed that our method successfully removed all visible mosquito-associated debris (Fig 1D). To quantitatively assess contaminants, samples were normalised by mosquito equivalents (MEQ); based on the number of mosquitoes (mq) homogenised and volume (units: mq/ml) as opposed to sporozoite dose, recognizing that this can vary substantially between batches. To assess the sequential reduction in protein contaminants during each step of the purification, uninfected mosquitoes were purified to determine contributing mosquito protein contaminants. MAF samples were run at three different MEQs (300, 100, and 50 mq/ml) on FFE to determine an optimal purification condition. Uninfected mosquitoes processed by MAF showed a complete absence in detectable mosquito-derived protein by silver stain when loaded onto FFE at 100 mq/ml or less. An equivalent preparation of the same number of MEQs using uninfected mosquito-derived SGD showed only a 63.1% reduction in contaminants when compared with crude input (Fig 1E). The differences in the FFE separation profile of mosquito-associated protein at the three MEQs demonstrated that at 100 mq/ml or less, all detectable protein could be effectively removed from the peak sporozoite positive FFE fractions (Fig 1F).

Analysis of the FFE output demonstrated our ability to remove abundant mosquito proteins, such as actin, as well as enriching for sporozoite proteins in the sporozoite fraction (Fig 1D–F). In addition, an identical protein purification profile was obtained when using dissected salivary glands as the starting homogenate rather than whole mosquitoes (referred to as Dissected-Accudenz-FFE; DAF) (Fig S1D), demonstrating the flexibility of our stepwise process for isolating different sub-populations of sporozoite within the infected mosquito, for example, where separation of immature haemocoel sporozoites from those in the head/salivary gland is desired.

**Figure S2.**
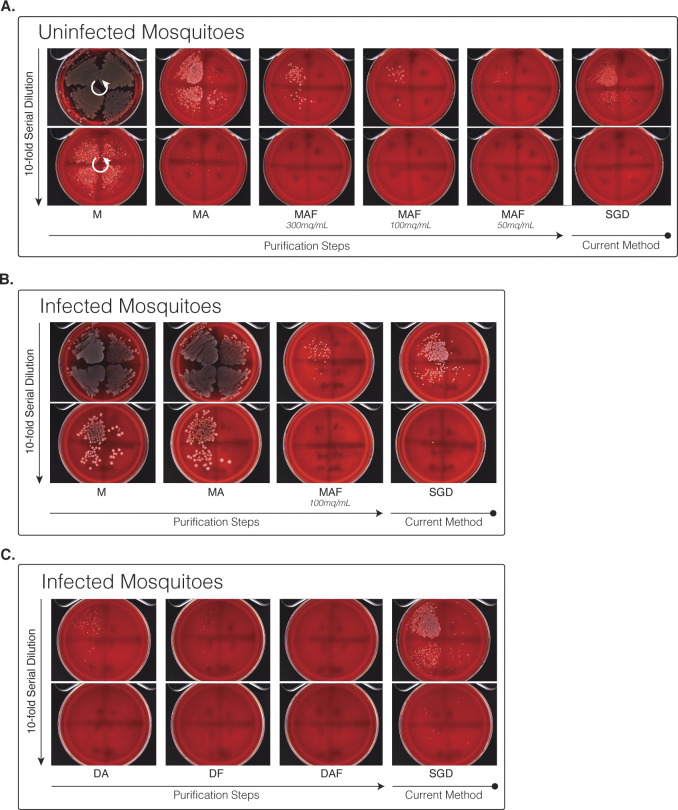
Blood plate agar growth of sporozoite purification steps. **(A)** Bacterial growth at different steps of purification from uninfected whole mosquito homogenate. Samples were loaded onto the free-flow electrophoresis machine at three different MEQs (300, 100, and 50 mq/ml). **(B)** Bacterial growth at different purification steps from infected whole mosquito homogenate. **(C)** Bacterial growth at different stages from infected dissected salivary gland homogenate. All samples spread onto blood agar plates in eight, 10-fold serial dilutions running anti-clockwise, with plates separated into four quadrants and dilutions starting in the top right quadrant (indicated by white arrows).

Given that bacterial contamination is a major problem for in vitro work, we next assessed the ability of our stepwise process to separate mosquito-associated bacteria. Serial dilutions of samples normalised to equal MEQ from each stage of purification were grown for 16 h at 37°C in a non-selective tryptic soya broth medium (50) (Fig 1G). A marked reduction in bacterial growth, assessed by measuring OD600, was observed with MAF purified sporozoites. This was further confirmed by measuring bacteria colony-forming units per ml (cfu/ml) on blood-agar plates, which showed a significant 8.1 log reduction in total bacterial load compared with a 5.9 log reduction by SGD (Figs 1H, S1G, and S2A–C). This translates to a >150-fold reduction in the bacterial load when compared to equivalent numbers of sporozoites obtained by SGD. Repeating the method with DAF produced sporozoites (dissected salivary glands used as input for FFE processing) demonstrated the successful removal of all detectable bacteria (Fig 1I).

**Figure S3.**
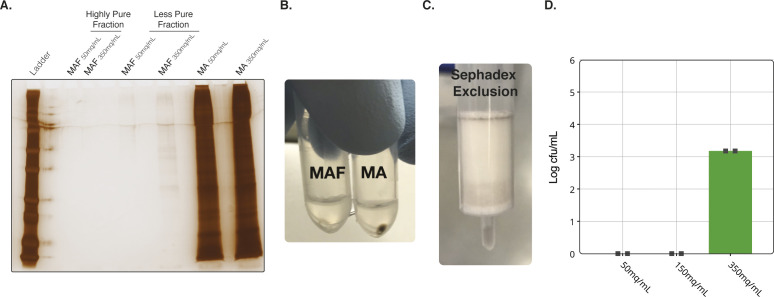
MAF Purification using Sephadex and interval zone electrophoresis methods. **(A)** Silver stain of each stage of purification illustrating the high- and low-purity fractions. All samples run at identical MEQs. **(B)** Images of pellets from MA and MAF purification. **(C)** Images of a Sephadex column after eluting sample. **(D)** Bacterial cfu/ml of samples injected into the free-flow electrophoresis (interval zone electrophoresis) at three concentrations of MEQs.

As an alternative to density gradients, we also tested whether rapid gel filtration with a Sephadex-based spin-column (5 min) could replace Accudenz, mirroring a method used with bovine sperm purification (51). Use of Sephadex resulted in sporozoite losses of <10% compared with Accudenz, which resulted in sporozoite losses of up to 50%. In parallel, we tested whether an interval zone electrophoresis (iZE) FFE method (see the Materials and Methods section) could add further improvements to our overall process (Fig S3A–D). Combining Sephadex with iZE, we were able to produce completely sterile sporozoites from whole mosquitoes (Fig S3D). However, because purity was associated with an additional substantial loss in yield (∼80%), the remainder of the development of the method (and experiments described below) used MAF purification and an FFE input of 100 mq/ml separated via cZE. Optimisation of Sephadex and iZE clearly has potential to further advancement of sterile sporozoite production at scale (important for good manufacturing process (GMP) licensure) and is the focus of future work.

### MAF sporozoites show improved in vitro infectivity compared with those from SGD

**Figure 2.**
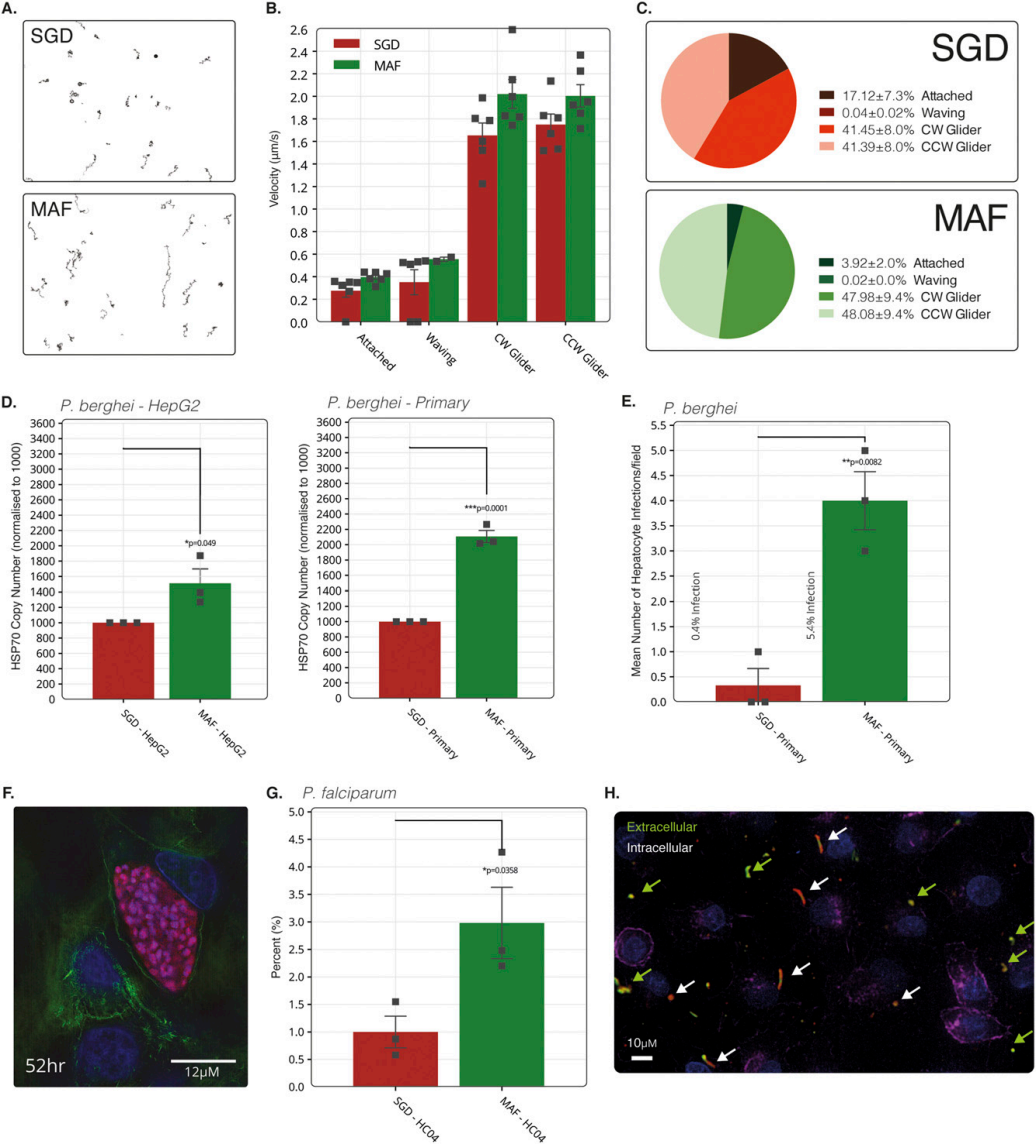
Assessment of purified sporozoite in vitro viability. **(A)** Typical movement trails of MAF and salivary gland dissection (SGD) sporozoites over 600 frames at 2 Hz. **(B)** Sporozoite gliding motility over 600 frames at 2 Hz with a sliding nine frame average during each motility state over 600 frames at 2 Hz. **(C)** Comparison of the percentage of all sporozoites in each state. Sporozoite tracking represents mean of two independent replicates and six technical replicates with groups compared using an unpaired two-tailed *t* test. Bars represent means and error bars the SEM. A total of 10,672 and 8,370 sporozoites were counted for SGD and MAF, respectively. **(D)** Absolute RT-PCR quantification of parasite HSP70 housekeeping gene DNA copies normalised by host HSP60 gene in HepG2 (left) and primary rat (right) hepatocytes. Treatments for both HepG2 and primary hepatocytes were normalised to 1,000 *hsp70* copies for the SGD treatment. Means of three independent replicates. **(E)** Mean counts of successful hepatocyte infections in primary rat hepatocytes measured by visual identification of six fields of view over 24-h time-lapse from three independent replicates. **(F)** Fluorescent image of late-stage schizont (52 h) captured using structured illumination microscope. Blue; nuclei, green; actin, red; mCherry parasite, pink; parasite actin (anti-5H3 [75]). **(G)** Means counts of initial hepatocyte invasions of *Plasmodium falciparum* sporozoites 4 h post infection in HC-04 at a ratio of 1:5 cells to sporozoites. SGD treatment normalised to 1. Sporozoites stained for CSP to determine intracellular or extracellular location. One independent replicate with three technical replicates. **(H)** Immunoflourescent staining of HC-04 cells with fixed 4 h after infection with *P. falciparum* sporozoites and stained with anti-CSP (extracellular = green + red, intracellular = red only), DAPI for nuclear material (blue) and phalloidin for actin (purple).

Using the devised stepwise method described (MAF), we next sought to assess the in vitro infectivity of sporozoites isolated in this way. Sporozoite motility is often used as a primary indicator of sporozoite viability (23). Comparisons of the motility patterns of SGD *P. berghei* sporozoites versus those from MAF on a glass surface revealed no significant differences in the 2D motion patterns displayed (static, attached/waving or gliding) (52), either in terms of mean velocity or overall ratios of motion pattern (Fig 2A–C).

**Figure S4.**
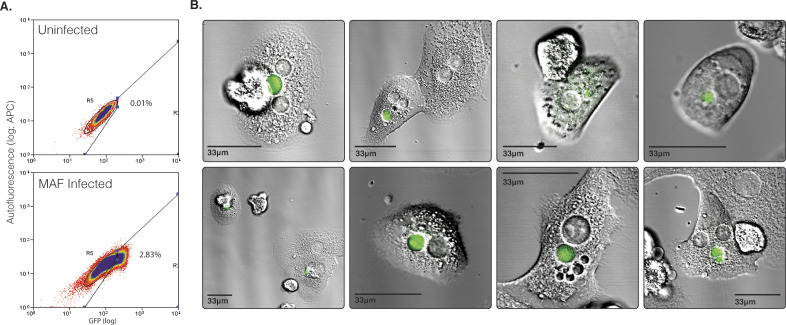
Ex-vivo development of MAF purified *Plasmodium berghei* sporozoites in rat primary hepatocytes. **(A)** The ability of MAF sporozoites to infect hepatocytes in vivo but develop ex vivo was investigated. Hepatocytes were extracted by perfusion of livers that were collected from rats 14 h after i.v. injection of sporozoites into rats. These rats were infected with a total of 3 × 10^7^ GFP-expressing *P. berghei* sporozoites purified by MAF (whole mosquitoes) from 400 mosquitoes. Infected hepatocytes from these rats were collected by flow-sorting and subsequently plated and incubated for a period of up to 30 h. Flow sorting identified 2.83% GFP-positive cells in the extracted, perfused liver cell population. **(B)** Fluorescent images of GFP-positive cells collected by flow sorting 24 h after plating.

**Figure S5.**
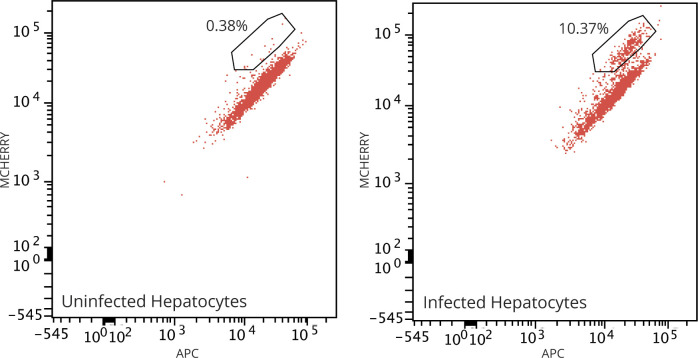
MAF *Plasmodium berghei* flow cytometry. Flow cytometry quantification of mCherry expressing transgenic *P. berghei* infected primary rat hepatocytes 36 h post addition of sporozoites (total 2,808 cells in treatment, 3,906 cells in control). Data representative of three technical replicates.

Extending infectivity analysis to in vitro infection of hepatoma or primary hepatocytes, RT-PCR analysis of *P. berghei* copy number, 24 h post infection (p.i.), showed a 1.5- and 2.1-fold increase in MAF sporozoite infectivity in HepG2 and primary rat hepatocytes when compared with that of SGD sporozoites, respectively (Fig 2D). Of note, the proportion of *P. berghei* exo-erythrocytic forms developing in primary rat hepatocytes 24 h p.i. was 13.5-fold increased when MAF sporozoites were used rather than their SGD counterparts (infection rate of 5.4% versus 0.4%) (Fig 2E). At 52 h p.i., MAF sporozoites had completed maturation into late schizonts, as indicated by the presence of liver-stage merozoites (Fig 2F). These sporozoites also fully developed into late-stage exoerythrocytic schizonts when infected hepatocytes were extracted from rats and cultured ex vivo (Fig S4). Corroborating these results, assessment of MAF infected rat primary hepatocytes by flow cytometry, showed infection rates of 10.4% (302 of 2,808 cells, 1:1 ratio of cells to sporozoites) (Fig S5). Mirroring observations in *P. berghei*, human-infective *P. falciparum–*derived sporozoites, processed through the same MAF process, were shown to have a threefold increased rate of invasion into HC-04 cells when compared to sporozoites isolated by SGD (Fig 2G and H). The difficulties of setting up a robust human primary hepatocyte model for long-term in vitro development with *P. falciparum* precluded our ability to take these to late stage schizogony. Overall, analysis of *P. berghei* and *P. falciparum* sporozoites purified by our stepwise method nonetheless demonstrates that they exhibit at least equivalent and potentially superior infectivity in vitro.

### MAF sporozoites from segmented mosquitoes are more infectious than SGD in vivo

**Figure 3.**
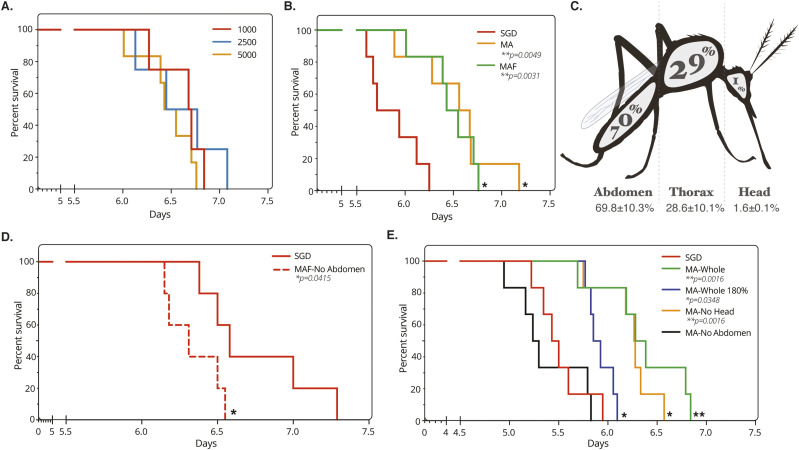
Assessment of purified sporozoite in vivo viability. **(A)** Kaplan–Meier survival curve of mice challenged i.v. with increasing doses of sporozoites from MAF. Six mice per group. End point classed as 1% parasitaemia. **(B)** Kaplan–Meier survival curve of mice challenged i.v. with 5,000 sporozoites from different purification steps. Six mice per group. End point classed as 1% parasitaemia, treatments compared by Mantel–Cox statistical test. **(C)** Sporozoite distribution of infected mosquitoes, average from 85 mosquitoes, two experimental replicates. Values show mean with SEM. Raw sporozoite numbers per mosquito are as follows: abdomen: 95,950; thorax: 42,500; head: 2,292. **(D)** Kaplan–Meier survival curve of mice challenged i.v. with 1,000 sporozoites from MAF-No Abdomens purified (MAF from mosquitoes with abdomens removed before homogenisation) and salivary gland dissection origin. Six mice per group. End point classed as 1% parasitaemia, treatments compared by Mantel–Cox statistical test. **(E)** Kaplan–Meier survival curve of mice challenged with 5,000 sporozoites obtained by MA purification from different mosquito sources or salivary gland dissection origin. Six mice per group. Death classed as 1% parasitaemia, treatments compared by Mantel–Cox statistical test.

As MAF sporozoites showed the potential for enhanced infectivity in vitro compared with SGD counterparts, further in vivo studies were performed to confirm this trend. Mice were inoculated with *P. berghei* sporozoites by i.v. injection and infectivity determined by measuring the time to reach 1% blood-stage parasitaemia (prepatent period), a standard measure of infectivity used in this field of research (53). Mice were inoculated i.v. with escalating doses of MAF purified *P. berghei* sporozoites, demonstrating that, independent of the inoculum size, MAF sporozoites were able to develop and establish a successful blood-stage infection (Fig 3A).

We next compared infectivity of MAF sporozoites with SGD sporozoites. Initially, 5,000 sporozoites obtained from either SGD, MA or MAF were given i.v. to mice with resulting blood-stage parasitaemia monitored. Partially purified MA sporozoites and fully purified MAF sporozoites showed a modest but significant delay in time to 1% parasitaemia compared to SGD (0.66 d longer for MA, **P = 0.0049; 0.59 d longer for MAF, **P = 0.0031; Mantel–Cox Test) (Fig 3B). This result was in contrast to in vitro infections, which were significantly increased with MAF sporozoites. It is clear that sporozoites purified from whole mosquitoes will necessarily include a proportion of immature haemocoel sporozoites that have yet to reach the salivary glands. We therefore reasoned that this reduction in in vivo infectivity could be due to the presence of less infective sporozoites in the MA and MAF inoculum. Indeed, whereas sporozoites obtained from the mosquito haemocoel can infect hepatocytes in vitro and in vivo (30), studies with salivary gland sporozoites have generally exhibited markedly increased in vivo virulence compared to less mature haemocoel sporozoites (30, 47). To investigate the relative proportion of immature midgut-derived/haemocoel sporozoites in whole mosquito homogenates, mosquitoes at 21 d post infectious bloodmeal were therefore separated into abdomen, thorax (containing the salivary glands) and head. Segmentation of the mosquito in this manner revealed that a significant number of sporozoites are found in the abdomen (70%) compared to the thorax (29%), which contains the salivary glands (Fig 3C).

To address whether inclusion of haemocoel sporozoites was responsible for reduced infectivity, we subtracted immature sporozoites from the initial inoculum by removing mosquito abdomens to assess whether this reverted the delay in time to patency. Mosquito abdomens were removed before the initial homogenisation step in our purification platform (MAF-No Abdomen) and, as a measure of sporozoite in vivo infectivity, the time to 1% blood parasitaemia was monitored as before. Notably, in mice infected with MAF-No Abdomen sporozoites there was now a marked increase in infectivity (time to 1% parasitaemia) when compared with SGD sporozoites (*P = 0.04; Mantel–Cox Test; Fig 3D). Of note, this increase in infectivity was only seen when sporozoites went through all steps of the purification pipeline, as no differences were observed between mice challenged with SGD and partially purified MA-No Abdomen sporozoites (MA with abdomens removed before homogenisation) (Fig 3E). This indicates that the FFE stage of the method is key to increasing sporozoite infectivity, while also showing that by increasing the dose of sporozoites extracted from whole mosquitoes with intact abdomens (i.e., accounting for the immature population in the haemocoel) it is possible to compensate for this delay (180%, 1.8-fold increase; Fig 3E). These data show that the purification method described can yield sporozoites with increased in vitro and in vivo infectivity; however, to observe this increase in infectivity directly in vivo, dose must be compensated for to account for immature haemocoel-derived sporozoites.

### Vaccination with irradiated MAF sporozoites confers sterile protection

**Figure 4.**
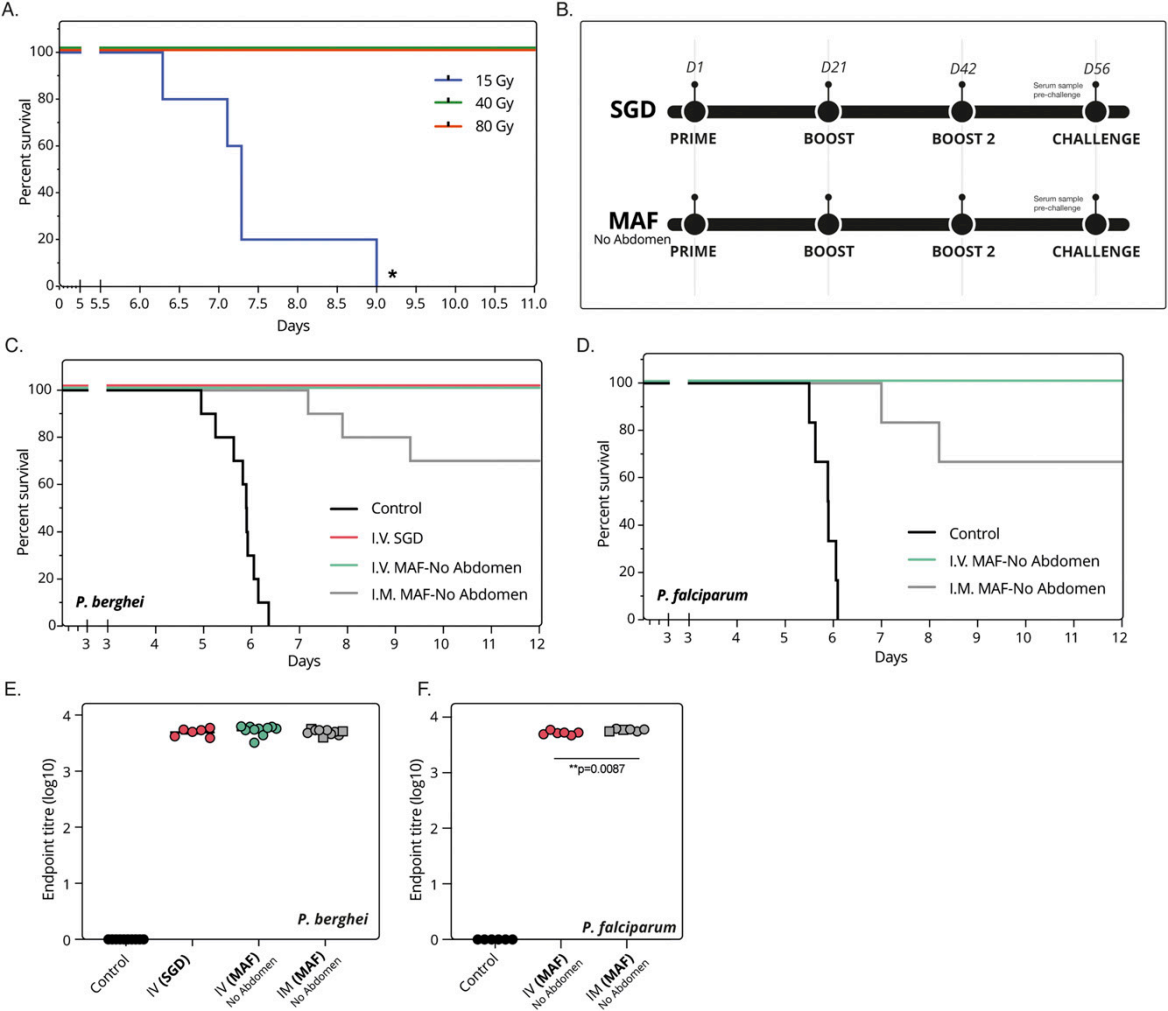
Purified sporozoites as a viable vaccine. **(A)** Kaplan–Meier survival curve of mice challenged i.v. with 1,000 *Plasmodium berghei* sporozoites from MAF-No abdomen purified and gamma irradiated. Four mice per group. End point classed as 1% parasitaemia. **(B)** Schematic of vaccination regime used. Sporozoites were either from salivary gland dissection or MAF-No Abdomen origin, then gamma irradiated. **(C)** Immunisation i.v. or i.m. of Balb/c mice with irradiated *P. berghei* sporozoites from either manual salivary gland (salivary gland dissection) dissection or MAF-No Abdomen. Mice given three immunisations of 40,000 sporozoites, 2 wk apart followed by challenge with five infectious mosquito bites. Ten mice per group. End point classed as 1% parasitaemia. **(D)** Immunisation i.v. or i.m. of Balb/c mice with irradiated *Plasmodium falciparum* sporozoites from MAF-No Abdomen. Mice given three immunisations of 40,000 sporozoites, 2 wk apart followed by challenge with five infectious mosquito bites. Six mice per group. End point classed as 1% parasitaemia. **(E)** Total titres of IgG antibodies against *P. berghei* sporozoite lysate in mouse serum before challenge **(F)** Total titres of IgG antibodies against *P. falciparum* sporozoite lysate in mouse serum before challenge. Squares indicate mice not protected.

Having developed a process that produces sporozoites with high purity and high infectivity, we next sought to assess the potential of MAF sporozoites as a radiation-attenuated sporozoite vaccine (RASv). To enable an effective comparison of efficacy with sporozoites of the same maturity to those from SGD (i.e., to account for immature haemocoel sporozoites for fair head-to-head comparison), we used MAF sporozoites from mosquitoes without abdomens for immunisation experiments. Before immunisation, the effective irradiation dose was determined to be 60 Gy by i.v. inoculation with varying doses of γ-irradiated sporozoites (Fig 4A). Mice were immunised i.v. using a three-immunisation regime of 40,000 irradiated sporozoites, 2 wk apart. In parallel, cohorts of control mice were immunised with plain medium as controls. Immunisation efficacy was assessed by challenging with five infectious mosquito bites (Fig 4B) (46). Immunisation with *P. berghei* (wild-type PbANKA) or *P. falciparum* (wild-type NF54) MAF-RASv sporozoites i.v. achieved complete protection against native *P. berghei* or chimeric *P. berghei* expressing *P. falciparum* CSP (PbANKA-PfCSP), respectively (Fig 4C and D). The level of protection (sterile protection) was comparable to that offered by SGD-RASv. Equivalent total IgGs measured against whole sporozoites pre-challenge were found in the serum from immunised animals irrespective of the source of *P. berghei* sporozoites (Fig 4E).

Finally, considering the practical development and utilisation of a whole sporozoite vaccine and the comparatively challenging nature of i.v. administration, we sought to address whether alternative routes of immunisation such as intramuscular (i.m.) might become possible given the shortened time to patency of MAF-No abdomen produced parasites. Previous attempts at i.m. immunisation using *P. berghei* demonstrated a level of protection around 30% (47). Intramuscular immunisation of 40,000 irradiated MAF sporozoites with two boosts was given with the commercial adjuvant AddaVax, a squalene-based oil-in-water nano-emulsion (InvivoGen). Sporozoites produced by our MAF method showed 70% and 67% protective efficacy for both *P. berghei* and *P. falciparum* MAF-RASv immunisation compared to non-immunized controls, respectively. Comparison of total IgG against sporozoites between i.v. and i.m revealed similar antibody titres for *P. berghei* immunisations (Fig 4E), whereas a modest increase in titre was observed with *P. falciparum* between i.m. and i.v. routes of immunisation (Fig 4F). Although direct comparison with data under different conditions from previous studies is challenging (47), it is clear that MAF-produced sporozoites show great potential for whole-sporozoite vaccination, which critically is not dependent on manual SGD.

## Discussion

We present here a robust stepwise method for the isolation of large quantities of pure malaria sporozoites that does not require manual SGD. Sporozoites isolated by this dissection-independent method exhibited improved sterility and enhanced infectivity in vitro when compared to SGD sporozoites, improved in vivo infectivity when compared with sporozoites of the same maturity (MAF without abdomens), and conferred sterile protection in a mouse challenge model. With demonstrated application to both rodent *P. berghei* and human-infective *P. falciparum*, sporozoite production using this process represents a potentially transformative technology that has multitude applications. Not least, this stepwise process can serve as an optimal starting point for development of a dissection-independent manufacturing process for GMP grade whole-sporozoite vaccines.

**Figure S6.**
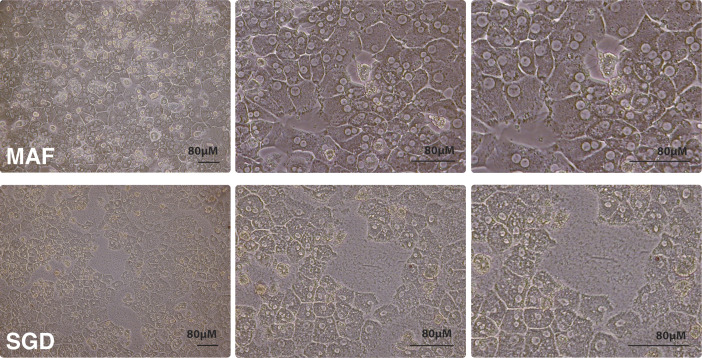
Sporozoite-associated morphological changes in primary rat hepatocytes. Bright-field images of primary rat hepatocytes 20 h after addition of sporozoites obtained by either MAF (top row) or salivary gland dissection (bottom row). Cultured in 1% P/S.

By combining bulk mosquito homogenisation, Sephadex filtration (or density centrifugation), and FFE separation (abbreviated to MAF), sporozoites purified using the stepwise method developed here could be obtained significantly faster than SGD and with all detectable mosquito-associated protein removed. In addition to the improved levels of purity gained, MAF sporozoites also showed a markedly improved infectivity in vitro for both rodent and human malaria parasites. The reduced overall time required and consistency of production are likely to be key factors in determining in vitro infectivity. However, several additional factors likely account for this improvement in infectivity. When MAF and SGD sporozoites were added to primary human hepatocytes, SGD treatment was found to be associated with abnormal human cell morphology and reduced cell numbers (Fig S6), suggesting mosquito contaminants may be detrimental to host-cell growth, reducing overall viability of hepatocytes. To account for this confounding variable seen with in vitro experiments, in vivo infection studies exploring whether our purified sporozoites directly exhibited improved infectivity compared with SGD sporozoites were undertaken with MAF sporozoites of comparable maturity (i.e., deriving from mosquitoes with abdomens removed). These sporozoites of comparable maturity showed improved infectiousness, suggesting that overall our process likely contributes not just to purity but also to improved infectiousness in vitro.

When exploring other possible reasons for improved infectivity we noted that MAF sporozoites had 4.3 times more cleaved CSP on their surface than SGD sporozoites (Fig S1D) (48, 49). Previous work has explored the importance of CSP processing on *P. berghei* sporozoite invasion. Of note, genetically altered sporozoites expressing a pre-cleaved CSP showed greater levels of in vitro infectivity (27). The proportion of CSP that is processed following MAF purification appears to be accelerated and therefore may be a contributing factor to improved infectivity of sporozoites isolated in this way, although this will require further experimental validation. Finally, a recent study identified the mosquito salivary protein, mosGILT as negatively modulating sporozoite motility (25). Other mosquito-associated factors that reduce hepatocyte infectivity may be forthcoming and would likely be purified away from sporozoites using the MAF protocol, which may further contribute to improved hepatocyte infectivity. Controlling these factors in the future may help advance in vitro liver-stage systems and high-throughput assays that yield relatively low numbers of infected hepatocytes after exposure to *P. falciparum* sporozoites (20, 54, 55). Ultimately, this should advance the development of comparable liver-stage platforms to those available for asexual blood-stage or sexual stage high throughput in vitro platforms (56). Further work is clearly warranted towards this.

Although MAF sporozoites showed the same motility patterns as SGD, this was somewhat unexpected given that MAF sporozoites originate from the entire mosquito. Sporozoites originating from less mature stages, such as those in oocysts or the haemolymph, are typified by reduced gliding motility compared to mature salivary-gland resident sporozoites (30, 47, 52). Indeed, we found that 70% of sporozoites in a typical day 21 post mosquito feed were abdominal in origin, conforming with previous observations (27) and indicating that most MAF sporozoites would be derived from less mature oocyst or haemolymph stages. Studies on the infectiousness of sporozoite developmental stages have shown that oocyst sporozoites (i.e., within the abdominal section) are more than 1,000-fold less infectious than salivary gland sporozoites by i.v. challenge (57). Thus, a significant proportion of injected sporozoites post-isolation may potentially be poorly infective, supporting a long-held belief that gliding per se may not be a good indicator of infectiousness. Our own infection data corroborate this, showing that use of whole mosquito homogenate was associated with a delay in blood-stage parasitaemia when compared with production via our process but with prior removal of the abdomen. Removal of the abdomen reverted any delay seen and demonstrated an increased time to patency compared to SGD (Fig 3D). Because removal of abdomens but without FFE (MA) did not advance infectivity (Fig 3E), this indicates that it is the FFE step that is critical to improving the viability of MAF sporozoites over SGD and not the removal of the abdomen on its own. Thus, efforts seeking to enrich specifically for highly infectious sporozoites (though not necessarily relevant for vaccination) may require prior abdomen removal from the mosquito before FFE for maximal infectivity. We note, however, that infectivity per se may not be the only requirement for overall efficacy in vaccination, indeed having a mixed population of immature sporozoites with those that are hyper-infective may be beneficial for broadly activating different arms of the immune system.

Sterile protection in mice is possible after administration of SGD sporozoites. Mirroring many similar studies, once irradiated, our MAF sporozoites showed full protection in both rodent and human challenge models via the i.v. immunisation route with similar antibody titres. Because it has recently been demonstrated that there are mosquito associated proteins that are able to modify the human immune response, it may be expected that such proteins could also interfere with the efficacy of a whole-sporozoite vaccines. Indeed, in agreement with this, recent work (58) showed that using Accudenz with SGD sporozoites to reduce total protein load was associated with improved pre-primed sporozoite (boost) specific CD8 T-cell responses compared with standard SGD. Thus, mosquito contaminants may cause innate immune up-regulation in vivo with unknown, if not confounding, effects on vaccination studies. Because MAF sporozoites showed a marked reduction in mosquito-associated protein and bacterial load, with potential for sterility using the iZE FFE method, this suggests that MAF sporozoites may well outperform SGD sporozoites in future head-to-head immunogenicity comparisons. Our ability to gain significant protection by i.m. immunisation route (60–70%) is certainly suggestive of markedly improved immunogenicity than previously obtained with SGD alone (47). Interestingly, recent work has shown intradermal immunisation with SGD-RASv was also associated with reduced efficacy compared with i.v. when challenged intradermally (59). Further work testing doses of sporozoites between MAF and SGD will be required to accurately assess comparative immunogenicity of different sporozoite sources and routes of immunisation.

In conclusion, the work presented here shows the development of a complete stepwise method for purification of large numbers of highly infectious sporozoites in a scalable format that is entirely compatible with basic biological, drug-screening, and whole-parasite vaccine studies. Our process yields sporozoites at higher purity compared with those from dissected preparations alone and is associated with a marked increase in in vitro hepatocyte infections and, once immature sporozoites from the abdomen are controlled for, enhanced in vivo infectivity. Sporozoites harvested by our process show markedly reduced levels of contaminants, can be produced aseptically and, critically, can be used to demonstrate high protective efficacy after both i.v. and intramuscular immunisation. For basic sciences, this stepwise method will be an important step towards single cell -omic studies that require large amounts of highly pure sporozoites, free from mosquito-associated contaminants that will unavoidably limit some of the scope or depth of coverage of such studies (60, 61). Concurrently, application and future adaptation of this technology to a GMP-compliant vaccine development pipeline, including genetically attenuated sporozoites (62, 63), offers the tantalizing opportunity to develop and manufacture pure, viable, immunogenic whole-parasite sporozoite vaccines at a dramatically increased scale when compared with current methods. Production of sporozoites at scale will no doubt advance our understanding of malaria liver-stage biology and help address the critical global need of an effective antimalarial vaccine.

## Materials and Methods

### Mosquito maintenance

*Anopheles stephensi* mosquitos used for experiments were raised at 28°C, 70% relative humidity with a 12-h light cycle. Larvae were fed with fish pellets and adults maintained on 10% fructose (reared by Alex Fyfe and Mark Tunnicliff).

### *P. berghei* maintenance and infection

**Figure S7.**
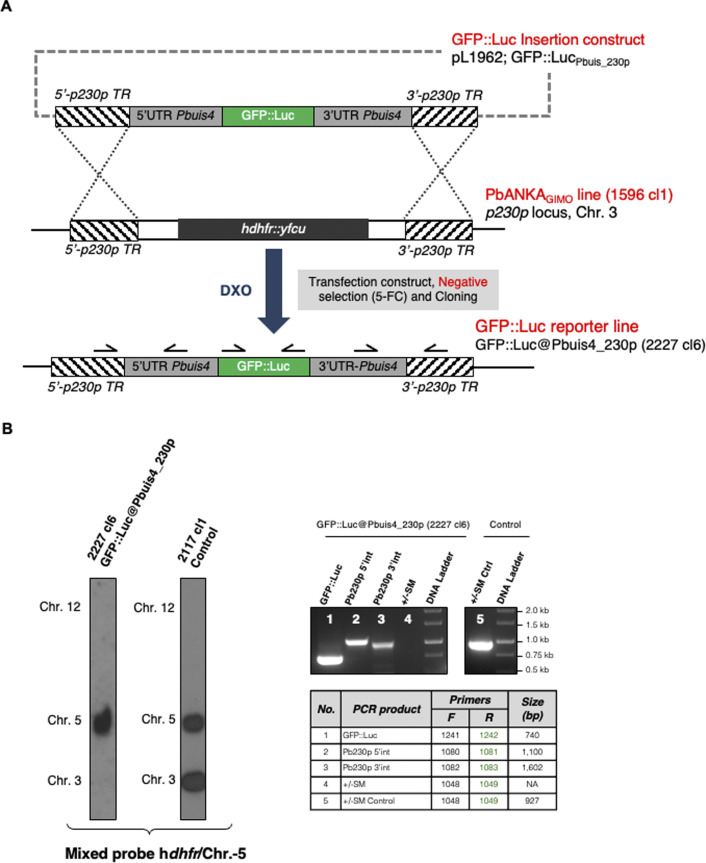
Generation and genotype analysis of the reporter line *PbANKA*-GFP::Luc@Pbuis4_230p. **(A)** Schematic representation of the introduction of the GFP::Luciferase expression cassette into the genome of the gene insertion/marker out PbANKA parent line 1596 cl1. The *gfp-luciferase* fusion gene is under the control of *Pbuis4* regulatory sequences (5′UTR and 3′UTR regions). DNA construct pL1962 containing the GFP::Luciferase expression cassette is integrated into the modified *Plasmodium berghei p230p* locus on chromosome (chr.) 3, containing the h*dhfr*::y*fcu* selectable marker (SM) cassette (black box), by double cross-over homologous recombination (DXO) at the *p230p* target regions (hatched boxes). Negative selection with 5-FC selects for parasites (line 2227 cl6) that have the GFP::Luciferase expression cassette introduced into the neutral *p230p* gene locus and the h*dhfr*::y*fcu* marker removed. Location of primers used for PCR analysis and sizes of PCR products are shown. See Tables S1 and S2 for primer sequences. **(B)** Conformation of correct integration of the GFP::Luciferase expression cassette into the genome by Southern analysis of pulsed-field gel–separated chromosomes and diagnostic PCR. Left panel: Hybridisation of pulsed-field gel–separated chromosomes (chr.) of the reporter line *PbANKA*-GFP::Luc@Pbuis4_230p shows integration of the expression cassette into the *p230p* gene insertion/marker out locus on chromosome (chr.) 3 by the absence of the h*dhfr*::y*fcu* SM cassette in the cloned reporter line. The Southern blot was hybridized with a mixture of two probes: one recognizing h*dhfr* and a control probe recognizing chr. 5. As an control parasite line, line 2117 cl1 was used with the h*dhfr*::y*fcu* SM integrated into chr. 3. Right panel: Diagnostic PCR shows the absence of the h*dhfr*::y*fcu* SM, the presence of GFP::Luciferase gene and the correct integration of the expression cassette into the genome of *PbANKA*-GFP::Luc@Pbuis4_230p at the 5′- and 3′-regions of *p230p* (5′int and 3′int). See (A) for the primers’ locations and Table S2 for primer sequences. Source data are available for this figure.

Two transgenic *P. berghei* ANKA lines were used in this study that express either mCherry or GFP under control of the *uis4* promoter. This promoter drives transgene expression specifically in sporozoites and liver stages. The transgene expression cassettes of both lines have been introduced into the neutral *p230p* gene locus by the method of gene insertion/marker out (GIMO) transfection (64). The generation and characterisation of the mCherry-expressing line mCherry@*Pbuis4*_230p (line 2204) has been described previously (65). The generation and characterisation of the GFP-expressing line GFP @*Pbuis4*_230p (line 2227) was generated as follows: The *P. berghei* ANKA line GIMO parent line 1596cl1 (64) was used for transfection with a construct (pL1962) which targets the neutral *p230p* locus (PBANKA_030600) and inserts GFP::Luciferase expression cassette, thereby removing the selectable marker (SM) consisting of human dihydrofolate reductase and the yeast cytosine deaminase and uridyl phosphoribosyl transferase (h*dhfr::yfcu*), according to the GIMO transfection technique, which has previously been described (64). The transfection vector, which lacks a drug SM cassette, was obtained using the using the standard GIMO DNA construct pL0043 (64). The expression cassette contained the GFP::Luciferase flanked by the 5′ and 3′ promoter and transcription terminator sequences of *P. berghei uis4* gene (PBANKA_0501200), which were amplified from *P. berghei* ANKA wild-type genomic DNA. The regulatory sequences of *uis4* gene were chosen to express GFP::Luciferase in sporozoites and liver stages (66, 67). Sequences of primers used for pL1962 construct generation are listed in Table S2. Transfection (exp. 2227) of 1596cl1 was performed using standard transfection methods (68) and negative selection was applied by treating mice with the 5-fluorocytosine (5-FC) in drinking water as described for GIMO transfection (64). The selected parasites were cloned by limiting dilution in mice and line 2227cl6 was further characterized for correct integration of GFP::Luciferase expression cassette into the *p230p* locus by diagnostic PCR and Southern analysis of pulsed-field gel electrophoresis–separated chromosomes (68). Sequences of primers used for PCR genotyping are listed in Table S3. The selected parasite 2227cl6, named GFP::Luc@Pbuis4_230p, contains the fusion gene *gfp-luciferase* under the control of the *uis4* regulatory sequences integrated into the neutral *p230p* locus and is SM free (see Fig S7A and B and source data). For vaccination studies mice were immunised with either the PbANKA 2.34 (wild type) or NF54 (wild type). Subsequent challenge was with either PbANKA 2.34 or PbANAKA 2.34 transgenic for *P. falciparum* CSP (PbANKA-PfCSP chimeric [69]).

Table S2 Primers for generation of pL1962 DNA construct.

Table S3 Primers for genotyping the GFP::Luc@Pbuis4_230p (2227 cl6) reporter line.

For infection of mice, cryopreserved parasitized RBCs (day 5) were thawed and injected into naïve Balb/c mice by the intraperitoneal (i.p.) route and *An. stephensi* mosquitos allowed to feed on anesthetised mice with 1–2% blood-stage parasitaemia. 7–10 d later, these mosquitoes were allowed to take an additional bloodmeal on naïve Balb/c mice to increase sporozoite yields. Blood-fed mosquitos were maintained at 19°C at 70% relative humidity for 19–22 d before sporozoites were extracted. Infection yielded an average salivary gland load of 40,000 sporozoites per mosquito.

### *P. falciparum* maintenance and infection

The wild-type NF54 *P. falciparum* strain was used in this study and cultured in vitro and gametocytes induced as per Delves et al (70). Briefly, asexual cultures were grown in RPMI 1460, supplemented with 25 mM Hepes (Life Technologies), 50 µg l^−1^ hypoxanthine (Sigma-Aldrich) and 10% A+ human serum (Interstate Blood-Bank). Gametocyte cultures were grown in RPMI 1640 supplemented with 25 mM Hepes (Life Technologies), 50 µg l^−1^ hypoxanthine (Sigma-Aldrich), 2 g l^−1^ sodium bicarbonate (Sigma-Aldrich), 5% A+ human serum (Interstate Blood-Bank), and 0.5% AlbuMAX II (Life Technologies). For standard membrane-feeding assays, 15–17 d-old gametocyte cultures were diluted in fresh RBCs and human serum at 50% haematocrit and used to feed female overnight-starved mosquitoes. Mosquitoes were maintained for 16–18 d before sporozoites were extracted. Infection yielded an average salivary gland load of 20,000 sporozoites per mosquito.

### Manual salivary gland dissection

Mosquitoes were sedated on ice for 10 min, then placed on a glass slide with 100 μl complete Schneider’s *Drosophila* medium (1% FBS, 4°C, NaHCO_3_ free, Pan-Biotech) and whole salivary glands removed by gentle separation of the head using micro-forceps. Both sets of glands were gently cleaned to remove other tissues and then placed into a glass Dounce tissue grinder on ice using 2 μl fresh medium. The glass slide was cleaned between each dissection. Each dissection took ∼45–90 s and was carried out for no more than 2–3 h maximum to reduce loss of infectivity. To release sporozoites the salivary glands were homogenised with three gentle but firm grinds using the pestle. The sample was transferred to protein lo-bind tubes (Eppendorf) used to prevent loss of sporozoites by adhesion to plastic-ware and mixed well before a sample was added to a haemocytometer and the average of four 16 square fields counted. Sample was diluted if too concentrated to accurately count.

### Homogenisation and accudenz gradient purification/Sephadex

Mosquitoes sedated on ice were placed in a Petri dish with 2 ml (per 400 mosquitoes) complete Schneider’s *Drosophila* media and gently homogenised with the end of a 10-ml syringe barrel for 30–60 s or using a gentleMACS homogeniser (Miltenyl Biotech). Liquid was removed and passed through a 100-μM cell strainer in a 50-ml centrifuge tube. A further 1.5 ml media was added to the petri dish, gently homogenised and passed through the 100-μM filter. This was repeated twice more but with more vigorous grinding. Finally, the filter was washed with 1 ml media. The filtrate was subsequently passed through a 70-, 40-, and 20-μM filter and each washed with 1 ml media (M). All steps were carried out on ice. On some occasions, the head and/or abdomens were removed before homogenisation. 1 ml homogenate was loaded onto a 3-ml Accudenz cushion (4°C) in a 15-ml centrifuge tube and centrifuged (2,500*g*, 4°C) as per reference 24.

Subsequently, 400 μl was taken from the sporozoite enriched boundary (at the 3 ml mark). 1 ml aliquots of Accudenz sporozoites were put into 2-ml protein lo-bind tubes (Eppendorf), made up to 2 ml with complete Schneider’s media and centrifuged (12,000*g*, 4°C, 3 min). The resultant pellet was re-suspended in complete Schneider’s media (MA). If the sample was to be used for FFE it was re-suspended to a mosquito equivalent (ME) of 200 mq/ml (mosquitoes/ml; unless stated otherwise), which was based on the original number of whole mosquitoes homogenised and the final volume this was in. The resultant sporozoite suspension was in most cases further purified by FFE.

Alternatively, SG-15 Sephadex medium was prepared in 3% sodium citrate at a 1:1 vol/vol ratio overnight. Subsequently a PD10 column was packed with 5 cm depth Sephadex and homogenate applied. This was centrifuged for 1–5 min at 46–413*g*. Eluted samples were subsequently applied to FFE.

### FFE purification

Before sporozoite extraction, the FFE machine (FFE Service GmbH) was setup for cZE using a 0.5-mm ZE spacer. A separation buffer of 10 mM triethanolamine (TEA), 10 mM glacial acetic acid (HAc), and 250 mM sucrose was used with a stabilisation buffer of 100 mM TEA, 100 mM HAc, and 250 mM sucrose injected into the separation chamber at 150/300 ml/h. Electrodes were kept in 100 mM TEA, 100 mM HAc, and 250 mM sucrose with a voltage of 900/750 V and current and power limit of 150 mA and 150 W, respectively. MA sample was mixed 1:1 with separation buffer (now at 100 mq/ml) and injected into the separation chamber at the cathode end at a rate of 1,600 μl/hr and fractions collected 14 min after injection started and stopped 14 min after sample finished. Fractions were collected at 4°C in 2 ml protein lo-bind deep-well plates (Eppendorf) containing 400 μL complete Schneider’s medium. The peak sporozoite fraction(s) was identified by a haemocytometer and centrifuged in 2 ml protein lo-bind tubes (max, 4°C, 3 min) and the pellet re-suspended in 100–500 μl complete Schneider’s media (MAF). To compare purification stages all samples were re-suspended to the same ME. FFE ME dose was calculated based on the volume collected in the peak fraction. Alternatively, the FFE machine was setup for iZE using a 0.2-mm spacer with identical separation buffers. Settings were 1,200 V, 150 mA, and 120 W with injection at 2,000 μl/h.

### Hepatocyte culture

Tissue culture plates (MatTek Corporation) were pre-coated overnight or using plasma-treatment with a 0.1 M bicarbonate buffer (pH9.4) (71) of collagen I, collagen IV, fibronectin, and laminin (1 μg/cm^2^; Sigma-Aldrich). Human HepG2 hepatoma cell lines were maintained in complete DMEM (10% FBS, 1% penicillin/streptomycin, 5% L-glutamine; Sigma-Aldrich) at 37°C with 5% CO_2_. A confluent monolayer was maintained using an 18G syringe needle. HC-04 cells were maintained in complete medium (DMEM supplemented with F12, 10% FBS, 1% penicillin/streptomycin, and 5% L-glutamine; Sigma-Aldrich). To obtain primary hepatocytes, male Wistar rats (Crl:CD(SD), strain 001) were anesthetised and a 21G cannula was inserted into the hepatic portal vein and secured/sealed using tissue adhesive (3M). Liver perfusion medium (37°C; Thermo Fisher Scientific) was pumped through the cannula at 10 ml/min using a peristaltic pump and once the liver started to lighten (within 30 s) the speed was adjusted to 20 ml/min. Subsequently the inferior vena cava was cut and over the next 5 min blocked two to three times and the pump increased to 40 ml/min. After successful perfusion, the media was exchanged for liver digest medium (37°C; Thermo Fisher Scientific) and the same blocking procedure carried out for 8 min. The liver was subsequently transferred quickly to 4°C complete DMEM on ice, the liver disrupted (using gentleMACS homogeniser), and the passed through 100-μM cell strainers. The cell suspension was washed twice (50*g*, 5 min, 4°C) with a final re-suspension into 19 ml complete DMEM and 20 ml sterile isotonic percoll (90% percoll, 10% 10× PBS) and centrifuged (1.06 g/ml, 100*g*, 10 min, 4°C) to remove debris and dead cells (percoll purification modified from reference 72). The pellet was washed in complete DMEM and used to seed plates. Importantly the plates were not moved for 30 min to allow the cells to adhere evenly across the plate. They were then transferred to an incubator (37°C, 5% CO_2_) for 1–2 h before medium was exchanged with serum-free hepatocyte growth medium (Promocell) which was exchanged every 12–15 h.

### Sporozoite motility assessment

Sporozoites were added to 37°C complete DMEM and centrifuged (413*g*, 4 min) in glass bottom tissue culture plates for sporozoites to sediment. Fluorescent images were captured at 2 Hz for 600 frames at 20× magnification. Motility was assessed using the ToAST ImageJ plugin (52).

### Rodent sporozoite challenge

*P. berghei* sporozoites were extracted from infected mosquitoes using one of the described methods and diluted in complete Schneider’s *Drosophila* medium (1% FBS, 4°C). Mice were placed in a 37°C heat-box for 10 min before injection of 50 μl i.v. into either lateral tail vein of restrained mice. From day 4–5, parasitaemia was monitored by Giemsa-stained thin-blood film until 3 d of positive smears were obtained, mice were then euthanized. 1,000 red blood cells were then counted from each slide over multiple fields of view to determine parasitaemia. Treatments were blinded to the user. Time to 1% parasitaemia was then calculated by linear regression. If parasites were not detected by day 14 the mice were euthanized.

### Rodent vaccination

Before immunisations sporozoites were diluted in Schneider’s *Drosophila* media to 80 × 10^4^/ml and irradiated using a Cs-137 γ irradiation source with a Gammacell 3000. For i.v. immunisations, sporozoites were diluted to 40 × 10^4^ sporozoites/ml in Schneider’s *Drosophila* medium and 100 μl injected per mouse. For intramuscular immunisations prep was diluted to 40 × 10^4^ sporozoites/ml of Schneider’s media and mixed with equal volumes of AddaVax adjuvant (InvivoGen). Balb/c mice were immunised intramuscular with 50 μl per site sporozoite/adjuvant mixture at 0, 3, and 5 wk. The mice were challenged with five PbANKA 2.34 or PbANKA-PfCSP chimeric infected mosquito bites 1 wk after booster immunisation by allowing the mosquitoes to feed on the abdomen of each mouse for 15 min. The salivary glands from all blood-fed mosquitoes have been dissected after the bites to confirm the presence of infective sporozoites. Since day 4 post challenge, the immunised mice were checked daily for the presence of *P. berghei* blood stages by microscopic examination of Giemsa-stained thin smears of tail blood and time to 1% parasitaemia determined by linear regression as above. The mouse was classified as negative for infection when no blood stages of parasite were present on day 14 after challenge.

### ELISAs

Sporozoite lysate was prepared by pelleting MAF purified sporozoites and flash freezing, before diluting in PBS and using to coat 96-well immunosorbent plates (NUNC MaxiSorp) overnight (1,500 sporozoites per well). Subsequently, liquid was removed and wells allowed to air dry before blocking with 1% BSA in PBS. Wells were subsequently incubated with mouse serum with starting dilutions of 1:50 or 1:100 in 0.01% Tween-PBS. Anti-mouse IgG secondary antibody conjugated to AP (Sigma-Aldrich) was added after five washes in 0.01% Tween-PBS. AP was quantified after five washes in 0.01% Tween-PBS using 4-nitrophenyl phosphate disodium salt hexahydrate (Sigma-Aldrich) measured at 405 nm absorbance.

### In vitro hepatocyte infection

In vitro *P. berghei* sporozoite infections were carried out on HepG2 or primary hepatocytes 24 h after plating. Cells were plated into either 48 or 24 well plates (MatTek Corporation) at seeding densities of 50,000 and 150,000 for HepG2 cells, respectively, and 75,000 and 200,000 for primary rat hepatocytes, respectively. 24-well plates (Glass bottom; MatTek Corporation) were used for microscopy and 48 wells for quantitative PCR. Sporozoites in 4°C complete Schneider’s *Drosophila* media were diluted in pre-warmed (37°C) complete DMEM (for HepG2; Sigma-Aldrich) or primary hepatocyte medium (for primary hepatocytes; Promocell) to achieve a desired ratio of sporozoite to hepatocyte (1:1 for qPCR; 50,000–75,000 sporozoite per well or 1:2 for microscopy; 75,000–100,000 sporozoite per well) and the culture media was exchanged with the sporozoite media. Cell cultures were carefully returned to the incubator to prevent swirling and an uneven distribution of sporozoites. Media was then no-longer exchanged for the remainder of the experiment.

For *P. falciparum* infections, HC-04 cells (media composition according to Yang et al [73]) were plated on 96 well plates (MatTek Corporation) coated with a 1 μg/cm^2^ mix of collagen, fibronectin, and laminin (see above) at a seeding density of 17,000 cells per well. Sporozoites were added to cells at a ratio of 5:1 (i.e., 85,000 sporozoites per well). The plates were immediately spun down for 5 min at 1,650*g*, before being returned to the incubator. After 4 h, the cells were washed once with PBS and fixed with 4% PFA.

### Ex vivo hepatocyte challenge

Rats were i.v. challenged with 30 million GFP transgenic sporozoites and 14 h later hepatocytes extracted by liver perfusion (above). Infected (GFP positive) hepatocytes were sorted (MoFlo) and plated for up to 30 h.

### Bacterial contaminant quantification

To assess the sterility of each purification step, tryptic soya broth (Oxoid) was inoculated with samples normalised by MEQ and absorbance at 600 nm measured after 16 h incubation at 37°C. Alternatively, samples normalised by MEQ were serially diluted in PBS and spread on blood-agar plates incubated overnight at 37°C. Negative growth was confirmed by a further 24-h incubation.

### Protein purity quantification

For Western blotting, sample was lysed using radioimmunoprecipitation assay buffer with protease inhibitor cocktail (Sigma-Aldrich), protein concentration normalised using Pierce BCA protein assay kit (Thermo Fisher Scientific), and sample loaded onto a 12% TGX SDS–PAGE gel using reducing Laemmli buffer and transferred by semi-dry transfer onto a polyvinylidene difluoride (PVDF) membrane (Bio-Rad Laboratories). *P. berghei* CSP protein was probed using the 3D11 monoclonal (48) and detected using HRP chemiluminescence. Total protein concentration of purified mosquito sample was assessed in SDS–PAGE gels using Pierce silver stain kit (Thermo Fisher Scientific) or in solution using a Pierce BCA protein assay kit (Thermo Fisher Scientific). Dot blots were conducted on FFE fractions by loading 200 μl of each fraction onto a multiscreen-IP plate (0.45 μM; Millipore) pre-activated with methanol and incubated overnight (4°C) before probing and detection of anti-mosquito actin (A2066; Sigma-Aldrich) similar to Western blotting using HRP and ECL. Alternatively, protein contaminants were assessed using liquid chromatography tandem-mass spectrometry (LC-MS/MS) with prior sample preparation in 6 M urea, 100 mM tris (pH 7.8), 5 mM dithiothreitol, and 20 mM iodoacetamide with subsequent trypsin digestion overnight and desalting. Mass spectrometry output data were analysed using the Mascot algorithm (V2.4) and UniProt database. All media used for protein assessment were protein free. Equivalent volumes were injected onto FFE and collected for each treatment and total protein in each fraction quantified.

### Flow cytometry

Flow cytometry was carried out using an LSRII (Becton Dickson). Hepatocytes were washed three times in 1× PBS and removed by gentle cell scraping. Hepatocytes were gated for single cell using FSC-H versus FSC-A and mCherry-*P. berghei*–infected cells detected in the PE-Texas Red channel by comparing to APC channel auto fluorescence. Uninfected hepatocytes were run as controls. GFP-expressing *P. berghei*–infected primary hepatocytes were sorted using a MoFlo cytometer (Beckman) gated for GFP-positive single cells.

### Immunofluorescent staining

Cells were fixed with 4% paraformaldehyde and permeabilised using 1% Triton X-100. Before antibody incubation cells were blocked with 1% BSA and then probed with primary and then secondary antibodies in 1% BSA for 1–2 h. Nuclear staining was carried using DAPI. For CSP in/out staining fixed cells were probed with CSP antibody before and after permeabilisation.

### Fluorescent microscopy

Imaging of mCherry fluorescent *P. berghei* infected primary hepatocytes was carried out using 1.5 mm glass bottom dishes/plates (Mattek) on a wide-field fluorescent microscope with LED fluorescence light source at 2 Hz using the Metamorph software package (Ludwig Institute). Infection numbers were determined by manual counting using the mCherry channel. Late-stage schizonts were captured using structured illumination microscopy with a Zeiss, Elyra (Imperial College London, FILM facility). *P. falciparum*–infected cells were imaged on a Nikon Eclipse Ti (Imperial College London, FILM facility). Image processing and analysis was automated by running a custom macro in Fiji. Quantification of intracellular versus extracellular parasites was carried by determining the area fraction (as a %) of CSP in and outside of hepatocyte. Parasites were classed as inside cells if the area fraction of CSP outside and inside was <10% and >80%, respectively. HC-04 numbers were quantified using nuclear count. Cell infection was calculated as the % ratio of intracellular parasites to HC-04 nuclei.

### Quantitative PCR

DNA was extracted from cultures using phenol–chloroform–isopropanol precipitation and re-suspended in molecular grade water. Nucleic acid concentration was determined using a Qubit fluorometer (Thermo Fisher Scientific). Quantification of *P. berghei* hepatocyte infection density based on absolute genome copies was determined using a standard curve plasmid containing a 271-bp fragment from murine heat shock protein (HSP) 60 (Ensembl: ENSMUST00000027123) housekeeping gene and a 176-bp fragment from *P. berghei* HSP70 gene (PBANKA_071190). 100 ng of DNA template was amplified using SsoAdvanced Universal SYBR green supermix (Bio-Rad Laboratories) run on a CFX Connect RT-PCR machine (Bio-Rad Laboratories) as per manufacturers standard protocol and parasite genome numbers determined using linear fit normalised to HSP60 housekeeping (HSP60 HepG2 F: GACCAAAGACGATGCCATGC, R: GCACAGCCACTCCATCTGAA; HSP60 Rat F: TGGAGAGGTCATCGTCACCA, R: CACAGCTACTCCATCTGAGAGT; HSP70 *P. berghei* F: AGGAATGCCAGGAGGAATGC, R: AGTTGGTCCACTTCCAGCTG).

### Animal research

All animal works in this study were carried out according to the Animals (Scientific Procedures) Act 1986 Amendment Regulations 2012 (SI 2012/3039) with approval from the University of Oxford and Imperial College London Ethical Review Committee (PPL 30/2889 Oxford, 70/8788 and PDA3EBA4A Imperial). The Office of Laboratory Animal Welfare Assurance for Imperial College covers all Public Health Service supported activities involving live vertebrates in the US (no. A5634-01). Rats and mice were kept in individually ventilated cages.

### Statistical analysis

Data were assessed for normality and equality of variance and used to determine the suitable statistical test as per John Tukey’s exploratory data analysis method (74). Parametric data were assessed using a *t* test and non-parametrically using a Mann–Whitney U test for single treatment comparisons. Multiple treatments were compared using a *t* test with Bonferroni correction. Kaplan–Meier curves were compared using the Mantel–Cox test.

## Data Availability

All data generated or analysed during this study are included in the manuscript and supporting files.

## Supplementary Material

Reviewer comments
