# Supplementary material for: Dissection-independent production of Plasmodium sporozoites from whole mosquitoes
Source: Life Sci Alliance. 2021 Jun 16;4(7):e202101094. doi: 10.26508/lsa.202101094 (PMC8321652; doi:10.26508/lsa.202101094)
Supplement: Supplementary file 1 [file LSA-2021-01094_TableS1.docx]

# Supplementary Table 1 - Abbreviations

| **Abbreviation** | **Meaning** |
| --- | --- |
| **M** | Mash/homogenised mosquitoes |
| **MA** | Mash/homogenised mosquitoes + Accudenz |
| **MAF** | Mash/homogenised mosquitoes + Accudenz + Free Flow Electrophoresis |
| **SGD** | Salivary Gland dissected sporozoites |
| **D** | Dissected salivary gland mash/homogenate |
| **DA** | Dissected salivary gland mash/homogenate + Accudenz |
| **DAF** | Dissected salivary gland mash/homogenate + Accudenz + FFE |
| **MQ** | Mosquito |
| **MEQ** | mosquito equivalents, based on the number of mosquitoes (mq) homogenised and volume (units: mq/mL) |
