# Supplementary material for: Dissection-independent production of Plasmodium sporozoites from whole mosquitoes
Source: Life Sci Alliance. 2021 Jun 16;4(7):e202101094. doi: 10.26508/lsa.202101094 (PMC8321652; doi:10.26508/lsa.202101094)
Supplement: Supplementary file 2 [file LSA-2021-01094_TableS2.docx]

# Supplementary Table 2 - Primers for generation of pL1962 DNA construct

| **DNA Construct** | **Primer No.** | **Primer Sequences *** | **Restriction Sites** | **Fragment**  **Size (bp)** | **Description** |
| --- | --- | --- | --- | --- | --- |
| **pL1962** | **7169** | tat**cctgcagg**GTGATAGTGTAGATTTTTTTGTTTGAC | SbfI | 1,519 | Pbuis4 5’-UTR promoter sequence, F |
|  | **7170** | ataagaat**gcggccgc**AGACGTAATAATTATGTGCTGAAAGG | NotI |  | Pbuis4 5’-UTR promoter sequence, R |
|  | **7171** | cg**gatatc**TATAATTCATTATGAGTAGTGTAATTCAG | EcoRV | 1,025 | Pbuis4 3’-UTR sequence, F |
|  | **7172** | ggcc**ggtacc**TTTCGCTTTAATGCTTGTCATC | KpnI |  | Pbuis4 3’-UTR sequence, R |
|  | **7295** | ataagaat**gcggccgc**GATCTATGAGTAAAGGAGAAGAAC | NotI | 2,448 | GFP::Luc, F |
|  | **7296** | CTAGAATTACACGGCGATCTTTCC | -- |  | GFP::Luc, R |

* Red color: Restriction site sequence
