# Supplementary material for: Dissection-independent production of Plasmodium sporozoites from whole mosquitoes
Source: Life Sci Alliance. 2021 Jun 16;4(7):e202101094. doi: 10.26508/lsa.202101094 (PMC8321652; doi:10.26508/lsa.202101094)
Supplement: Supplementary file 3 [file LSA-2021-01094_TableS3.docx]

# Supplementary Table 3 - Primers for genotyping the GFP::Luc@Pbuis4_230p (2227 cl6) reporter line

| **Primer No.** | **Description** | **Primer sequence** |
| --- | --- | --- |
| **1048** | hDHFR-yFCU (+/-SM) F | ATCATGCAAGACTTTGAAAGTGAC |
| **1049** | hDHFR-yFCU (+/-SM) R | CATCGATTCACCAGCTCTGAC |
| **1080** | 5'-*p230p* Integration F | ACTGTTATATTTGGTGATGGAATGG |
| **1081** | 5'-*p230p* Integration R | TATACATCCACGGATGCATAGAAG |
| **1082** | 3'-*p230p* Integration F | TCTGCATTAACCTTAAATATGAAAAACAC |
| **1083** | 3'-*p230p* Integration R | TTCAGTGAAATCGCAAACATAAGTATC |
| **1241** | GFP::Luc F | ccgg**ggtaccctcgag**ATGAGTAAAGGAGAAGAACTTTTCACTG |
| **1242** | GFP::Luc R | cgA**GGATCC**CTTTGTATAGTTCATCCATG |

* Red/blue color: Restriction site sequence
