# Supplementary material for: Dissection-independent production of Plasmodium sporozoites from whole mosquitoes
Source: Life Sci Alliance. 2021 Jun 16;4(7):e202101094. doi: 10.26508/lsa.202101094 (PMC8321652; doi:10.26508/lsa.202101094)
Supplement: Reviewer comments [file LSA-2021-01094_review_history.pdf]

Life Science Alliance

Dissection-independent production of a Plasmodium sporozoites from whole mosquitoes

Joshua Blight, Katarzyna Sala, Erwan Atcheson, Holger Kramer, Aadil El-Turabi, Eliana Real, Farah Dahalan, Paulo Bettencourt, Emma Dickinson-Craig, Eduardo Alves, Ahmed Salman, Chris J Janse, Frances M Ashcroft, Adrian VS Hill, Arturo Reyes-Sandoval, Andrew Blagborough and Jake Baum

DOI: https://doi.org/10.26508/lsa.202101094

Corresponding author(s): Prof. Jake Baum (Imperial College London)

Review Timeline:

Submission Date:	2021-04-14
Editorial Decision:	2021-05-19
Revision Received:	2021-05-24
Accepted:	2021-05-25

Scientific Editor: Shachi Bhatt

Transaction Report:

(Note: With the exception of the correction of typographical or spelling errors that could be a source of ambiguity, letters and reports are not edited. The original formatting of letters and referee report s may not be reflected in this compilation.)

Please note that the manuscript was reviewed at Review Commons and these reports were taken into account in the decision-making process at Life Science alliance.

No full Peer Review Process File is available with this article, as the authors have chosen not to make the review process public in this case.

May 19, 2021

RE: Life Science Alliance Manuscript #LSA-2021-01094-T

Dr. Jake Baum
Imperial College London
Department of Life Sciences
South Kensington
South Kensington
London, London SW72Az

Dear Dr. Baum,

Thank you for submitting your revised manuscript entitled "Dissection-independent production of a Plasmodium sporozoites from whole mosquitoes". We would be happy to publish your paper in Life Science Alliance.

For a brief overview, the manuscript was previously reviewed at Review Commons and the authors transferred a revised version, along with the reviewers' comments, and their rebuttal to the reviewers' comments to Life Science Alliance (LSA). LSA editors found the findings intriguing and the authors' revisions sufficient and the paper is ready to be published in LSA, pending minor edits to meet the journal's formatting guidelines.

-please consult our manuscript preparation guidelines https://www.life-science-alliance.org/manuscript-prep and make sure your manuscript sections are in the correct order
-please upload your main and supplementary figures as single files
-please add a Category, a Summary Blurb/Alternate Abstract, and a Running Title for your manuscript in our system
-please add an Author Contributions in our system as well
-please rename "COMPETING INTERESTS" to "Conflict of Interest"
-please add your main, supplementary figure, and table legends to the main manuscript text after the references section
-we encourage you to check the panels in Figure S5 and update callouts and legend accordingly
-please upload your Supplementary Tables in editable .doc or excel format
-please use capital letters in callouts and legends and actual figures when introducing the panels, Fig 1a-> Fig 1A
-please add callouts for Figures S2A-C; S3A-D to your main manuscript text
-please add scale bars for Figure 1D
-please provide full gels and source data for the southern blots shown in Figure S7B

If you are planning a press release on your work, please inform us immediately to allow informing our production team and scheduling a release date.

To upload the final version of your manuscript, please log in to your account:

https://lsa.msubmit.net/cgi-bin/main.plex
You will be guided to complete the submission of your revised manuscript and to fill in all necessary information. Please get in touch in case you do not know or remember your login name.

To avoid unnecessary delays in the acceptance and publication of your paper, please read the following information carefully.

A. FINAL FILES:

These items are required for acceptance.

-- An editable version of the final text (.DOC or .DOCX) is needed for copyediting (no PDFs).

-- High-resolution figure, supplementary figure and video files uploaded as individual files: See our detailed guidelines for preparing your production-ready images, https://www.life-science-alliance.org/authors

-- Summary blurb (enter in submission system): A short text summarizing in a single sentence the study (max. 200 characters including spaces). This text is used in conjunction with the titles of papers, hence should be informative and complementary to the title. It should describe the context and significance of the findings for a general readership; it should be written in the present tense and refer to the work in the third person. Author names should not be mentioned.

B. MANUSCRIPT ORGANIZATION AND FORMATTING:

Full guidelines are available on our Instructions for Authors page, https://www.life-science-alliance.org/authors

We encourage our authors to provide original source data, particularly uncropped/-processed electrophoretic blots and spreadsheets for the main figures of the manuscript. If you would like to add source data, we would welcome one PDF/Excel-file per figure for this information. These files will be linked online as supplementary "Source Data" files.

Submission of a paper that does not conform to Life Science Alliance guidelines will delay the acceptance of your manuscript.

It is Life Science Alliance policy that if requested, original data images must be made available to the editors. Failure to provide original images upon request will result in unavoidable delays in publication. Please ensure that you have access to all original data images prior to final submission.

The license to publish form must be signed before your manuscript can be sent to production. A link to the electronic license to publish form will be sent to the corresponding author only. Please take a moment to check your funder requirements.

Reviews, decision letters, and point-by-point responses associated with peer-review at Life Science Alliance will be published online, alongside the manuscript. If you do want to opt out of having the reviewer reports and your point-by-point responses displayed, please let us know immediately.

Thank you for your attention to these final processing requirements. Please revise and format the manuscript and upload materials within 7 days.

Thank you for this interesting contribution, we look forward to publishing your paper in Life Science Alliance.

Sincerely,

Shachi Bhatt, Ph.D.
Executive Editor
Life Science Alliance
http://www.lsajournal.org
Tweet @SciBhatt @LSAjournal

May 25, 2021

RE: Life Science Alliance Manuscript #LSA-2021-01094-TR

Prof. Jake Baum
Imperial College London
Department of Life Sciences
Exhibition Road
South Kensington
London SW7 2AZ
United Kingdom

Dear Dr. Baum,

Thank you for submitting your Research Article entitled "Dissection-independent production of a Plasmodium sporozoites from whole mosquitoes". It is a pleasure to let you know that your manuscript is now accepted for publication in Life Science Alliance. Congratulations on this interesting work.

The final published version of your manuscript will be deposited by us to PubMed Central upon online publication.

Your manuscript will now progress through copyediting and proofing. It is journal policy that authors provide original data upon request.

Reviews, decision letters, and point-by-point responses associated with peer-review at Life Science Alliance will be published online, alongside the manuscript. If you do want to opt out of having the reviewer reports and your point-by-point responses displayed, please let us know immediately.

IMPORTANT: If you will be unreachable at any time, please provide us with the email address of an alternate author. Failure to respond to routine queries may lead to unavoidable delays in publication.

Scheduling details will be available from our production department. You will receive proofs shortly before the publication date. Only essential corrections can be made at the proof stage so if there are any minor final changes you wish to make to the manuscript, please let the journal office know now.

DISTRIBUTION OF MATERIALS:
Authors are required to distribute freely any materials used in experiments published in Life Science Alliance. Authors are encouraged to deposit materials used in their studies to the appropriate repositories for distribution to researchers.

You can contact the journal office with any questions, contact@life-science-alliance.org

Again, congratulations on a very nice paper. I hope you found the review process to be constructive and are pleased with how the manuscript was handled editorially. We look forward to future exciting submissions from your lab.

Sincerely,

Shachi Bhatt, Ph.D.
Executive Editor
Life Science Alliance
http://www.lsajournal.org
Tweet @SciBhatt @LSAjournal
